# Treatment Outcome of Surgical Protocols for Peri‐Implantitis: A Retrospective Cohort Study in a Specialised University Centre

**DOI:** 10.1111/jcpe.70115

**Published:** 2026-03-15

**Authors:** Wenjie Zhou, Ausra Ramanauskaite, Clemens Raabe, Emilio A. Cafferata, Neelam Lingwal, Frank Schwarz

**Affiliations:** ^1^ Department of Oral Surgery, Implantology and Oral Medicine Goethe University Frankfurt am Main Germany; ^2^ Second Dental Center, Ninth People's Hospital, Shanghai Jiao Tong University, School of Medicine Shanghai China; ^3^ Department of Oral Surgery and Stomatology School of Dental Medicine, University of Bern Bern Switzerland; ^4^ Oral Peri‐Implant Research Group, School of Dentistry, Universidad Científica del Sur Lima Peru

**Keywords:** disease resolution, peri‐implantitis, surgical treatment, treatment success

## Abstract

**Aim:**

To explore the treatment outcome of surgical peri‐implantitis protocols in a university‐based setting.

**Materials and Methods:**

Four‐hundred and six implants in 223 patients were surgically treated by four trained surgeons using open‐flap debridement (OFD, 37 implants), implantoplasty (Impl, 39 implants), reconstructive surgery (Rec, 241 implants) or a combined approach (Comb, 89 implants). Treatment success after 1 year (maximum probing depth (max PD) ≤ 5 mm, bleeding on probing (BOP) at ≤ 1 site and no suppuration) was the primary outcome. Logistic regression was used to explore associations between patient‐/implant‐related factors, treatment modalities and treatment success, implant loss and surgical retreatment.

**Results:**

Treatment was successful in 54.7% of implants after 1 year: OFD 62.5%, Impl 58.3%, Rec 55.4%, Comb 48.3%, with no significant group differences. Over a mean follow‐up of 30.80 ± 19.62 months, 40 implants (9.9%) were lost and 38 (9.4%) required surgical retreatment. In exploratory multivariable analyses, systemic antibiotic use (pre‐operative: OR = 3.54, *p* = 0.04; pre‐ and post‐operative: OR = 4.49, *p =* 0.02) and surgeon experience (OR = 0.12, *p* = 0.003) showed associations with treatment success. Molar site (OR = 3.21, *p* = 0.03), antibiotic (pre‐ and post‐operative: OR = 3.13, *p* = 0.02) and baseline suppuration (OR = 4.51, *p* = 0.002) were associated with implant loss. Surgical retreatment was associated with overdenture (OR = 3.59, *p* = 0.003) and max PD at baseline (OR = 1.30, *p* = 0.001).

**Conclusions:**

Surgical peri‐implantitis outcome was associated with systemic antibiotic, surgeon, molar site, overdenture, baseline suppuration and PD.

## 1 Introduction

Given its high prevalence, peri‐implantitis has become a major clinical challenge in implant dentistry, requiring effective treatment strategies to prevent disease progression and maintain the affected implant in function (Romandini et al. 2021; Obreja et al. 2021; Derks and Tomasi 2015). While non‐surgical approaches have shown limited and inconsistent efficacy in re‐establishing peri‐implant tissue health, surgical interventions are often implemented (Ramanauskaite, Fretwurst, and Schwarz 2021; Ramanauskaite, Galarraga‐Vinueza, et al. 2021).

Various surgical techniques—including open‐flap debridement, adjunctive implantoplasty or reconstructive therapy and combined approaches—have been proposed for disease management (Ramanauskaite, Fretwurst, and Schwarz 2025; Ramanauskaite, Saltzer, et al. 2025). The main goal of surgical peri‐implantitis treatment is to re‐establish peri‐implant tissue health and ensure the long‐term stability of both soft and hard tissues (Sanz and Chapple 2012; Herrera et al. 2023). Beyond resolving inflammation, reconstructive procedures aim to regenerate bone defects, achieve re‐osseointegration and maintain stable tissue levels post surgery (Jepsen et al. 2019).

Despite the availability of multiple surgical techniques, treatment success is still difficult to predict. Recurrent or persistent peri‐implant inflammation and ongoing bone loss are not uncommon findings, sometimes necessitating additional surgical re‐intervention or even implant removal (Schwarz et al. 2022; Karlsson et al. 2023; Donos et al. 2023). A multitude of patient‐ and implant‐related factors were shown to be associated with treatment outcomes, including the extent of initial bone loss, defect characteristics, implant surface characteristics, smoking status and patient compliance with maintenance care (Koldsland et al. 2018; Carcuac et al. 2017; Berglundh, Wennström, and Lindhe 2018; Monje et al. 2023; Ramanauskaite, Fretwurst, and Schwarz 2021; Ramanauskaite, Galarraga‐Vinueza, et al. 2021; de Waal et al. 2016; Ichioka et al. 2023; Ravidà et al. 2022).

Another underappreciated variable in the current literature is the heterogeneity of operator experience and treatment settings across studies. The majority of studies report on data from multiple centres (university clinics and private practices) and include surgeons of varying expertise, introducing additional factors that can confound the true efficacy of a given surgical protocol (de Waal et al. 2016; Ravidà et al. 2022; Alibegovic et al. 2025; Romandini et al. 2024; Derks et al. 2022). In light of these limitations, this study aimed to assess the treatment outcome of surgical peri‐implantitis treatments, performed by trained surgeons in a single academic centre, by analysing short‐ to medium‐term outcomes and identifying factors influencing treatment success.

## 2 Materials and Methods

### 2.1 Study Population

For this retrospective cohort analysis, two investigators (W.Z. and C.R.) screened standardised clinical records of patients who received surgical peri‐implantitis therapy at the Department of Oral Surgery and Implantology, Frankfurt, Germany, between April 2018 and June 2023. All patients were enrolled in an yearly supportive recall programme. The study protocol adhered to the principles of the Helsinki Declaration (as revised in 2013) and was approved by the local ethics committee (registration number: 2025‐2337). The study reporting follows the STROBE statement checklist (von Elm et al. 2008).

For patient selection, the following inclusion criteria were defined:

1. Presence of at least one screw‐type implant diagnosed with peri‐implantitis.
2. Implants affected by peri‐implantitis surgically treated.
3. Treated chronic periodontitis and regular periodontal maintenance care.
4. Patients attending at least 6 months of follow‐up appointments.
5. Complete clinical documentation.

Exclusion criteria included the following:

1. Uncontrolled systemic disorders affecting bone metabolism (diabetes, osteoporosis, arthritis, etc.).
2. Patients treated as part of clinical studies investigating innovative treatment approaches.
3. Zirconia implants.
4. Machined surface implants.

### 2.2 Treatment Procedures

Four trained oral surgeons performed all surgeries in line with the centre's protocols (preoperative modalities and antibiotic regimens are detailed in Table S1). In brief, under local anaesthesia, full‐thickness mucoperiosteal flaps were raised buccally and orally. After removal of granulation tissues with a curette, implant surfaces were decontaminated using a titanium brush (Hans Korea Co. Ltd.). The treatment modalities included the following adjunctive approaches:

- Open flap debridement (OFD):Flap repositioned to initial position.
- Implantoplasty (Impl):For horizontal bone loss, using diamond burs and Arkansas stones with sterile saline irrigation.
- Reconstructive therapy (Rec):Intrabony defects were treated using one of the following grafting materials: deproteinised bovine bone mineral (Bio‐Oss, Geistlich); collagen‐stabilised deproteinised bovine bone mineral (Bio‐Oss Collagen, Geistlich); or autogenous bone, each applied with or without a collagen membrane (Bio‐Gide, Geistlich).
- Combined treatment (Comb):For combined defects (horizontal + intrabony), implantoplasty plus one of the aforementioned reconstruction protocols.

All mucoperiosteal flaps were repositioned and adapted using non‐resorbable sutures. Post‐operative care included 0.12% chlorhexidine mouthwash twice daily for 5 days. Sutures were removed after 10 days of transmucosal healing.

### 2.3 Assessment of Clinical Outcome Measures

The following clinical measurements were recorded at baseline (i.e., prior to surgery, T0) and after 6 months (T1), 1 year (T2), 2 years (T3), 3 years (T4), 4 years (T5) and 5 years (T6) using a periodontal probe, and were extrapolated from the patient files by the treating surgeons:

(i) plaque (PI) (presence/absence), (ii) BOP (presence/absence), (iii) Sup (presence/absence), (iv) mean PD (measured from the mucosal margin to the bottom of the pocket), (v) max PD (the deepest PD value among implant sites), (vi) soft‐tissue recession (MR) (measured from the restoration margin to the mucosal margin), (vii) mean width of keratinised mucosa (KM) on the buccal aspect.

Except for KM, all parameters were recorded at six sites per implant: mesio‐buccal (mb), mid‐buccal (b), disto‐buccal (db), mesio‐oral (mo), mid‐oral (o), and disto‐oral (do). KM was measured only at the three buccal sites (mb, b, db).

- Primary outcome:

Treatment success at 1 year (T2) was defined based on three different combination criteria:

– Criterion 1: max PD ≤ 5 mm, ≤ 1 BOP site and no Sup (Berglundh, Armitage, et al. 2018);
– Criterion 2: reduction in mean PD, ≤ 2 BOP sites and no Sup;
– Criterion 3: reduction in max PD, ≤ 2 BOP sites and no Sup.

Implants lost at any timepoint after baseline were classified as having an unsuccessful outcome, regardless of peri‐implant clinical parameters at the time of implant removal.

- Secondary outcomes:
  – Mean values and changes in clinical parameters at baseline and follow‐up visits.
  – Implant loss and the need for surgical retreatment.

Along with the clinical parameters, the following patient‐ and implant‐site‐related data were extrapolated from patient files: age, gender, smoking status, general health status, jaw, region, implant type and prosthesis type.

### 2.4 Statistical Analysis

Statistical analyses were conducted using SPSS (version 19.0; IBM Corp.). The implant was the unit of analysis. Mixed‐effects models with patient‐level random intercepts were applied to account for within‐patient correlation due to repeated measurements. Binary outcomes were analysed using mixed‐effects logistic regression, whereas continuous variables were analysed using mixed‐effects quantile (median) regression to accommodate non‐normal data distribution. Post hoc comparisons between treatment groups at each time point were adjusted for multiple testing and false discovery rate (FDR).

Group comparisons between surgical procedure groups were performed using mixed‐effects logistic regression models with patient‐level random intercepts to account for clustering of implants within patients. Pairwise post hoc comparisons were performed for each criterion–time combination, with *p*‐values adjusted separately within each combination using the FDR method.

Regression analyses were conducted to examine associations between predictors and binary outcomes, including implant loss, treatment success at 1 year and the need for surgical retreatment, which served as dependent variables. Only variables found to be statistically significant in univariate screening were entered into the multivariable models as an initial variable reduction strategy. Multivariable analyses were fitted using mixed‐effects logistic regression models with patient‐level random intercepts. Model fit was assessed through null and residual deviance. Odds ratios (ORs) and 95% confidence intervals (CIs) were derived from the model coefficients. For outcomes with small sample sizes or potential separation issues, Firth logistic regression without random effects was additionally performed as a sensitivity analysis. Models included only complete cases, and model performance and convergence diagnostics were evaluated.

No a priori sample size calculation was performed because of the retrospective nature of the study. A post hoc power analysis was conducted based on the number of implants available at the 1‐year follow‐up, which constituted the dataset for the primary outcome analyses (total *n* = 274). This number included both implants remaining in situ at 1 year (*n* = 263) and implants lost within the first year (*n* = 11), which were classified as treatment failures and therefore also contributed to the primary endpoint. Using the observed effect size (Cramér's *V* = 0.1035) and a chi‐squared test (df = 3, *α* = 0.05), the estimated post hoc power was 27%, indicating limited ability to detect small‐to‐moderate between‐group differences. Because implants within the same patient are correlated, the effective statistical power after accounting for clustering is expected to be lower.

## 3 Results

### 3.1 Study Population and Treatment Modalities

**FIGURE 1:**
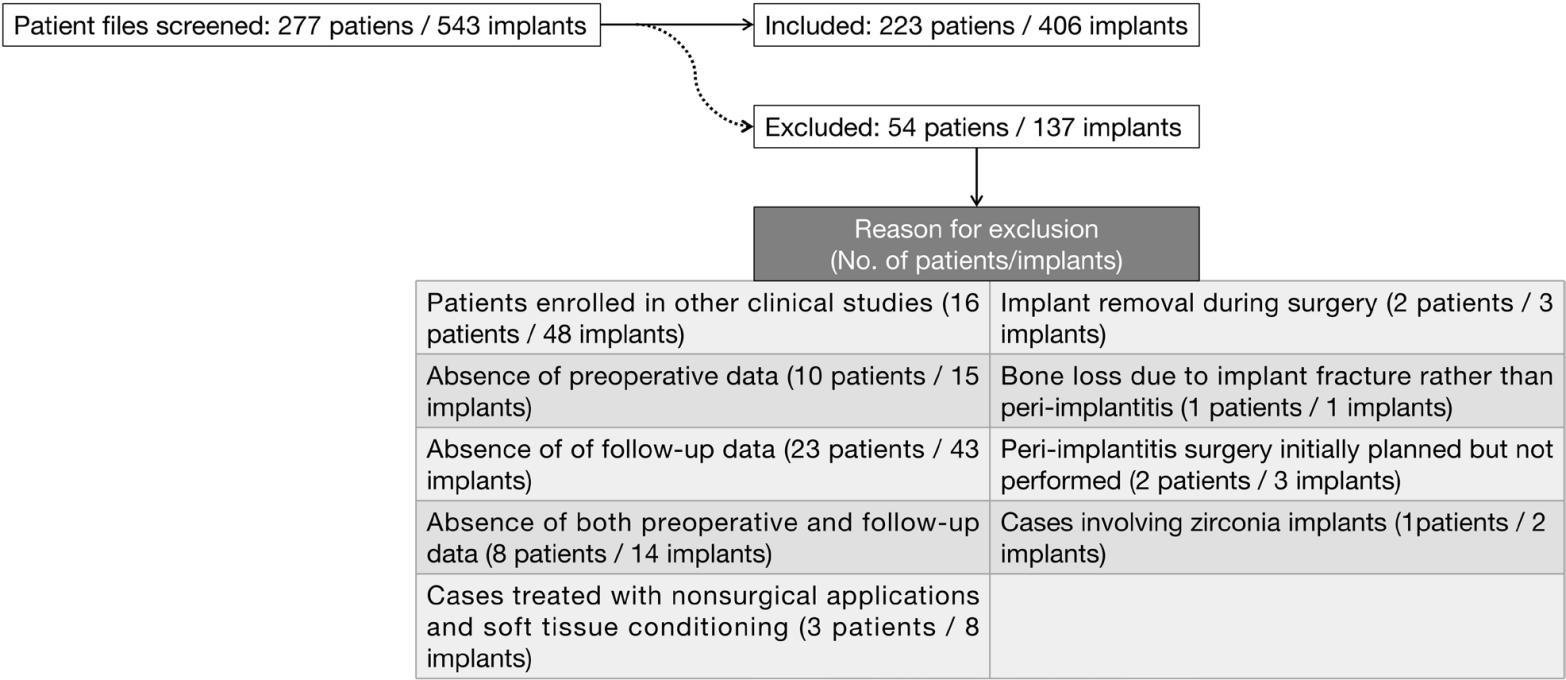
Flow‐chart depicting patient selection.

Of the 277 patient files with 543 implants screened, 223 patients with 406 implants were included in the analysis. The patient selection process is illustrated in Figure 1. Patient demographics and implant‐site characteristics are summarised in Tables 1 and 2. Sixty‐six patients with 94 implants were included in the previous analyses (40 patients/59 implants [Ramanauskaite, Fretwurst, and Schwarz 2025, Ramanauskaite, Saltzer, et al. 2025] and 26 patients/35 implants [Schwarz et al. 2023]). Treatment protocols are detailed in Table 3.

**TABLE 1:** Descriptive patient‐related data.

		Overall (*n* = 223 patients, %)
Age (years)		63.42 ± 12.86
Gender (male/female)		92/131 (41.3%/58.7%)
Smoking habit	Non‐smoker	184 (82.5%)
	Light smoker (< 10 cig./day)	17 (7.6%)
	Heavy smoker (≥ 10 cig./day)	22 (9.9%)
Other general diseases	None	123 (55.2%)
	Diabetes	4 (1.8%)
	Cardiovascular disease	67 (30.0%)
	Osteoporosis	13 (5.8%)
	Combined	16 (7.2%)
Medications	None	82 (36.8%)
	Antidiabetic agents (Insulin and Metformin)	9 (4.0%)
	Cardiovascular medications (antihypertensives and cardiovascular preventatives)	58 (26.0%)
	Anti‐osteoporotic agents (bisphosphonates)	6 (2.7%)
	Vitamin D supplements	9 (4.0%)
	Thyroid hormone replacement therapy	41 (18.4%)
	Acid‐suppressive medications (e.g., PPIs, H2 blockers)	5 (2.2%)
	Hormonal agents (e.g., glucocorticoids, oestrogens)	7 (3.1%)
	Antidepressants (selective serotonin reuptake inhibitors [SSRIs], e.g., paroxetine, fluoxetine)	2 (0.9%)
	Other bone‐active medications (e.g., IL‐inhibitor, RANKL inhibitor, methotrexate)	4 (1.8%)

**TABLE 2:** Descriptive implant‐related data.

			Overall (*n* = 406 implants, %)	OFD (*n* = 37 implants, %)	Impl (*n* = 39 implants, %)	Rec (*n* = 241 implants, %)	Comb (*n* = 89 implants, %)
Implant site	Region	Anterior	85 (20.9%)	8 (21.6%)	9 (23.1%)	51 (21.2%)	17 (19.1%)
		Premolar	156 (38.4%)	20 (54.1%)	24 (61.5%)	77 (32.0%)	35 (39.3%)
		Molar	165 (40.6%)	9 (24.3%)	6 (15.4%)	113 (46.9%)	37 (41.6%)
	Jaw	Maxilla	244 (60.1%)	25 (67.6%)	18 (46.2%)	153 (63.5%)	48 (53.9%)
		Mandible	162 (39.90%)	12 (32.43%)	21 (53.85%)	88 (36.51%)	41 (46.07%)
Implant and prosthesis type	Implant type	Bone level	359 (88.4%)	36 (97.3%)	32 (82.1%)	208 (86.3%)	83 (93.3%)
		Tissue level	47 (11.6%)	1 (2.7%)	7 (18.0%)	33 (13.7%)	6 (6.7%)
	Prosthetic type	Single crown	166 (40.9%)	11 (29.7%)	10 (25.6%)	115 (47.7%)	30 (33.7%)
		Bridge	145 (35.7%)	12 (32.4%)	13 (33.3%)	81 (33.6%)	39 (43.8%)
		Full‐arch fixed prosthesis	16 (3.9%)	1 (2.7%)	0 (0.0%)	11 (4.6%)	4 (4.5%)
		Overdenture	79 (19.5%)	13 (35.1%)	16 (41.0%)	34 (14.1%)	16 (18.0%)
			1 Locator; 2 stud; 12 bar; 64 telescope	3 bar; 10 telescope	4 bar; 12 telescope	1 bar; 2 stud; 31 telescope	1 Loactor; 4 bar; 11 telescope
Follow‐up after surgical peri‐implantitis treatment (months)			30.80 ± 19.62	25.24 ± 17.94	30.28 ± 17.74	30.18 ± 19.75	35.25 ± 20.23

**TABLE 3:** Treatment modality.

		Overall (*n* = 406 implants, %)	OFD (*n* = 37 implants, %)	Impl (*n* = 39 implants, %)	Rec (*n* = 241 implants, %)	Comb (*n* = 89 implants, %)
Antibiotics	None	145 (35.7%)	29 (78.4%)	21 (53.9%)	82 (34.0%)	27 (30.3%)
	Pre‐operative‐Ab	132 (32.5%)	13 (35.1%)	10 (25.6%)	85 (35.3%)	24 (27.0%)
	Post‐operative‐Ab	56 (13.8%)	6 (16.2%)	4 (10.3%)	25 (10.4%)	21 (23.6%)
	Pre‐ and post‐operative‐Ab	73 (18.0%)	3 (8.1%)	4 (10.3%)	49 (20.3%)	17 (19.1%)
Non‐surgical treatment before surgery	None	53 (13.1%)	3 (8.1%)	12 (30.8%)	23 (9.5%)	15 (16.9%)
	Er: YAG laser	194 (47.8%)	17 (46.0%)	14 (35.9%)	135 (56.0%)	28 (31.5%)
	Air‐flow	90 (22.2%)	9 (24.3%)	10 (25.6%)	48 (19.9%)	23 (25.8%)
	Chitosan brush	11 (2.7%)	0 (0.0%)	0 (0.0%)	5 (2.1%)	6 (6.7%)
	Mechanical debridement	51 (12.6%)	4 (10.8%)	2 (5.1%)	28 (11.6%)	17 (19.1%)
	Combined	7 (1.7%)	4 (10.8%)	1 (2.6%)	2 (0.8%)	0 (0.0%)
Membrane		—	—	—	154 (63.9%)	58 (65.2%)
Bone grafting materials	Autogenous bone	—	—	—	21 (8.7%)	7 (7.9%)
	Deproteinised bovine bone mineral	—	—	—	18 (7.5%)	7 (7.9%)
	Deproteinised bovine bone mineral + autogenous bone	—	—	—	81 (33.6%)	49 (55.1%)
	Collagen‐stabilised deproteinised bovine bone mineral	—	—	—	94 (39.0%)	24 (27.0%)
	Collagen‐stabilised deproteinised bovine bone mineral + autogenous bone	—	—	—	27 (11.2%)	2 (2.3%)

*Note*: –: not available.

### 3.2 Treatment Success

**FIGURE 2:**
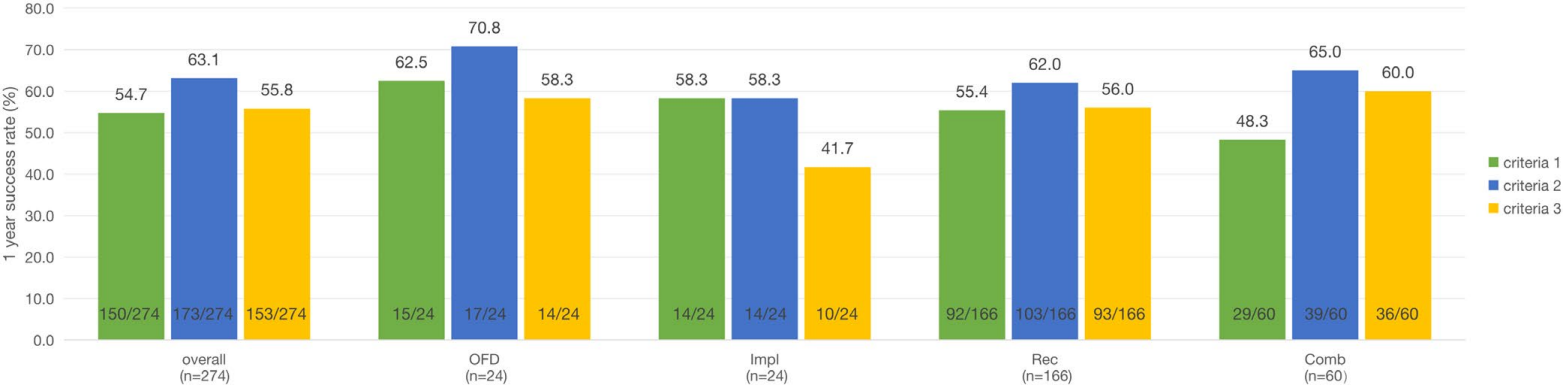
Bars represent the percentage of successful implants according to criteria 1–3 at T2. Numbers on top of bars indicate success rates (%). Fractions at the bottom inside each bar indicate the number of successful implants relative to the total implants in each group (*x*/*n*).

As shown in Figure 2, the 1‐year (T2) success rates according to the three specified criteria (criteria 1–3) did not differ significantly among the four treatment modalities (*p* > 0.05).

Treatment success at different follow‐up time points (Table S2) was highest at T1: Criterion 1, 59.1% (192/325); Criterion 2, 62.5% (203/325); Criterion 3, 57.5% (187/325). At T6, overall success rates were 26.9% (21/78), 29.5% (23/78) and 28.2% (22/78) for criteria 1–3, respectively. No significant differences were observed among surgical modalities for Criterion 1 and Criterion 3 at any time point. For Criterion 2, OFD (78.6%) and Rec (58.3%) both outperformed Impl (20.0%) at T3 (*p* = 0.02 and 0.03, respectively). No other significant differences were detected across groups or time points.

### 3.3 Implant Loss and Retreatment

Over a mean follow‐up period of 30.80 ± 19.62 months, 40 (9.9%) implants were lost in 30 patients. Non‐surgical retreatment was required in 133 (32.8%) implants in 85 patients, with a mean of 1.86 re‐treatment sessions per implant. Surgical retreatment was performed in 38 (9.4%) implants in 22 patients after a mean of 29.16 ± 19.88 months following the initial surgery.

Implant loss and the need for retreatment stratified by the surgical treatment modality are summarised in Table 4. Although the Comb group exhibited a higher rate of implant loss compared to the other modalities, this difference was not statistically significant (*p* = 0.13). However, retreatment—particularly non‐surgical—was more frequent in the Comb group, resulting in numerically higher retreatment rates compared with the other modalities. Although a nominal statistical significance was observed for overall retreatment (*p* = 0.02) and non‐surgical retreatment (*p* = 0.01), these findings should be interpreted cautiously because of limited sample size and group imbalance.

**TABLE 4:** Implant loss and the need for re‐treatment stratified by surgical approach.

		Overall (*n* = 406 implants)	OFD (*n* = 37 implants)	Impl (*n* = 39 implants)	Rec (*n* = 241 implants)	Comb (*n* = 89 implants)	*p*
Implant loss (%)		40 (9.9%)	3 (8.1%)	3 (7.7%)	19 (7.9%)	15 (16.9%)	0.13
Time to implant loss after the surgery (months)		27.38 ± 19.07	14.33 ± 5.77	29.33 ± 8.62	28.47 ± 25.28	28.20 ± 11.69	0.40
Need for retreatment	Overall (non‐surgical + surgical)	150 (37.0%)	7 (18.9%)	14 (35.9%)	86 (35.7%)	43 (48.3%)	0.02*
	Nonsurgical	133 (32.8%)	5 (13.5%)	12 (30.8%)	77 (32.0%)	39 (43.8%)	0.01*
	Surgical	38 (9.4%)	3 (8.1%)	3 (7.7%)	23 (9.5%)	9 (10.1%)	0.96
Time to surgical retreatment after the first surgery (months)		29.16 ± 19.88	28.33 ± 31.75	32.00 ± 29.10	31.74 ± 20.17	21.89 ± 12.82	0.69

* The difference is statistically significant among the four surgical modalities.

### 3.4 Clinical Parameters

Table 5 presents the mean value of the assessed clinical parameters at different time points, and Table 6 presents the change in the clinical parameters at T1–T6 compared to T0. Overall, all four treatment modalities showed comparable trends in changes in clinical parameters over time, with no significant differences detected among groups (*p* > 0.05) (Figures S1 and S2).

**TABLE 5:** Clinical parameters (mean, SD) measured at baseline (T0), after 6 (T1) months, 1 year (T2), 2 years (T3), 3 years (T4), 4 years (T5) and 5 years (T6).

	Baseline (T0)	OFD *n* = 37 implants	Impl *n* = 39 implants	Rec *n* = 241 implants	Comb *n* = 89 implants	*p*	6 months (T1)	OFD *n* = 29 implants	Impl *n* = 26 implants
	Overall *n* = 406 implants						Overall *n* = 319 implants		
PI, %	50.8%	48.7%	59.0%	46.5%	59.8%	A: 0.91 B: 0.97 C: 0.53 D: 0.98 E: 0.93 F: 0.46	38.6%	44.8%	53.9%
BOP, %	90.8%	91.9%	87.2%	91.3%	90.8%	A: 0.85 B: 1.00 C: 1.00 D: 0.65 E: 0.72 F: 1.00	33.9%	37.9%	42.3%
Sup, %	39.9%	43.2%	23.1%	40.7%	43.7%	A: 0.56 B: 1.00 C: 0.84 D: 0.32 E: 0.13 F: 0.75	3.5%	0.0%	0.0%
Mean PD, mm	5.06 ± 1.75	4.74 ± 1.59	4.39 ± 1.66	5.20 ± 1.78	5.10 ± 1.70	A: 0.69 B: 0.50 C: 0.69 D: 0.17 E: 0.29 F: 0.95	3.44 ± 1.36	3.28 ± 1.65	2.94 ± 0.83
Max PD, mm	6.82 ± 2.15	6.54 ± 2.06	6.00 ± 2.06	6.95 ± 2.15	6.97 ± 2.16	A: 0.74 B: 0.72 C: 0.72 D: 0.56 E: 0.56 F: 0.92	4.61 ± 1.89	4.52 ± 1.68	3.96 ± 1.11
MR, mm	0.41 ± 0.73	0.90 ± 0.84	0.82 ± 0.98	0.26 ± 0.62	0.43 ± 0.66	A: 0.59 B: < 0.001* C: 0.01* D: < 0.001* E: 0.10 F: 0.004*	0.66 ± 0.91	0.87 ± 0.81	1.31 ± 1.24
KM, mm	2.54 ± 2.02	2.24 ± 2.31	1.45 ± 1.71	2.75 ± 2.02	2.58 ± 1.86	A: 0.28 B: 0.34 C: 0.52 D: 0.003* E: 0.01* F: 0.81	2.68 ± 1.62	2.56 ± 1.64	1.62 ± 1.71

	Rec *n* = 193 implants	Comb *n* = 71 implants	*p*	1 year (T2)	OFD *n* = 22 implants	Impl *n* = 24 implants	Rec *n* = 159 implants	Comb *n* = 58 implants	*p* value
				Overall *n* = 263 implants					
PI, %	33.2%	45.1%	A: 0.98 B: 1.00 C: 0.95 D: 0.97 E: 1.00 F: 0.83	39.5%	54.6%	66.7%	35.9%	32.8%	A: 0.76 B: 0.60 C: 0.32 D: 0.05 E: 0.05 F: 0.76
BOP, %	28.0%	45.1%	A: 0.99 B: 0.86 C: 0.78 D: 0.61 E: 0.96 F: 0.04*	32.3%	22.7%	37.5%	32.7%	32.8%	A: 0.45 B: 0.40 C: 0.33 D: 0.99 E: 1.00 F: 0.98
Sup, %	3.6%	5.6%	A: 1.00 B: 1.00 C: 1.00 D: 1.00 E: 1.00 F: 0.85	1.9%	0.0%	4.2%	2.5%	0.0%	A: 1.00 B: 1.00 C: 1.00 D: 0.95 E: 1.00 F: 1.00
Mean PD, mm	3.51 ± 1.41	3.51 ± 1.22	A: 1.00 B: 0.42 C: 0.57 D: 0.28 E: 0.42 F: 0.95	3.39 ± 1.28	3.08 ± 1.69	3.44 ± 1.88	3.42 ± 1.02	3.41 ± 1.46	A: 0.69 B: 0.42 C: 0.69 D: 0.95 E: 1.00 F: 0.92
Max PD, mm	4.69 ± 2.01	4.63 ± 1.85	A: 0.88 B: 0.88 C: 0.92 D: 0.58 E: 0.72 F: 0.92	4.51 ± 1.71	4.05 ± 1.94	4.71 ± 2.27	4.52 ± 1.45	4.60 ± 2.02	A: 0.71 B: 0.58 C: 0.72 D: 0.92 E: 0.92 F: 0.92
MR, mm	0.47 ± 0.81	0.85 ± 0.91	A: 0.43 B: 0.003* C: 0.67 D: 0.002* E: 0.27 F: 0.002*	0.65 ± 0.88	1.14 ± 0.88	1.10 ± 0.95	0.46 ± 0.77	0.79 ± 0.98	A: 0.95 B: 0.002* C: 0.17 D: 0.002* E: 0.24 F: 0.02*
KM, mm	2.93 ± 1.58	2.42 ± 1.52	A: 0.14 B: 0.46 C: 0.82 D: 0.003* E: 0.13 F: 0.15	2.66 ± 1.52	2.32 ± 1.62	2.18 ± 1.71	2.85 ± 1.44	2.45 ± 1.57	A: 0.82 B: 0.27 C: 0.81 D: 0.15 E: 0.61 F: 0.28

2 year (T3)	OFD *n* = 11 implants	Impl *n* = 14 implants	Rec *n* = 110 implants	Comb *n* = 46 implants	*p*	3 year (T4)	OFD *n* = 8 implants	Impl *n* = 11 implants	Rec *n* = 85 implants	Comb *n* = 31 implants	*p*
Overall *n* = 181 implants						Overall *n* = 135 implants					
47.5%	63.6%	57.1%	49.1%	37.0%	A: 0.81 B: 0.57 C: 0.25 D: 1.00 E: 0.83 F: 0.59	41.5%	50.0%	54.6%	41.2%	35.5%	A: 0.93 B: 0.92 C: 1.00 D: 1.00 E: 0.84 F: 0.58
35.9%	0.0%	71.4%	32.7%	41.3%	A: 1.00 B: 1.00 C: 1.00 D: 0.07 E: 0.48 F: 0.38	41.5%	37.5%	27.3%	41.2%	48.4%	A: 0.88 B: 1.00 C: 0.91 D: 0.61 E: 0.33 F: 0.79
6.6%	0.0%	7.1%	3.6%	15.2%	A: 1.00 B: 1.00 C: 1.00 D: 0.85 E: 0.93 F: 0.07	7.4%	0.0%	0.0%	10.6%	3.2%	A: 1.00 B: 1.00 C: 1.00 D: 1.00 E: 1.00 F: 0.93
3.69 ± 1.44	3.50 ± 1.44	3.57 ± 1.36	3.66 ± 1.27	3.70 ± 1.68	A: 0.95 B: 0.92 C: 0.95 D: 0.95 E: 1.00 F: 0.95	3.70 ± 1.28	3.75 ± 1.97	3.39 ± 0.97	3.80 ± 1.28	3.47 ± 1.12	A: 1.00 B: 0.95 C: 1.00 D: 0.83 E: 1.00 F: 0.69
5.02 ± 1.95	4.64 ± 1.96	4.36 ± 1.65	5.01 ± 1.73	5.07 ± 2.14	A: 0.92 B: 0.83 C: 0.92 D: 0.65 E: 0.74 F: 0.92	5.05 ± 1.75	5.12 ± 2.53	4.55 ± 1.44	5.25 ± 1.68	4.52 ± 1.52	A: 0.92 B: 0.92 C: 0.92 D: 0.72 E: 0.92 F: 0.58
0.76 ± 1.00	0.89 ± 0.88	1.77 ± 1.53	0.55 ± 0.81	0.95 ± 1.03	A: 0.27 B: 0.27 C: 0.95 D: 0.02* E: 0.20 F: 0.06	0.69 ± 0.93	1.35 ± 1.19	1.36 ± 1.18	0.50 ± 0.80	0.82 ± 0.95	A: 0.91 B: 0.21 C: 0.32 D: 0.07 E: 0.29 F: 0.27
2.96 ± 1.55	3.18 ± 1.80	2.48 ± 1.86	3.09 ± 1.55	2.75 ± 1.42	A: 0.52 B: 0.82 C: 0.54 D: 0.52 E: 0.82 F: 0.38	3.08 ± 1.72	2.42 ± 1.76	1.79 ± 2.01	3.38 ± 1.66	2.90 ± 1.60	A: 0.62 B: 0.38 C: 0.65 D: 0.13 E: 0.28 F: 0.52

4 year (T5)	OFD *n* = 6 implants	Impl *n* = 11 implants	Rec *n* = 44 implants	Comb *n* = 27 implants	*p*	5 year (T6)	OFD *n* = 3 implants	Impl *n* = 2 implants	Rec *n* = 26 implants	Comb *n* = 13 implants	*p*
Overall *n* = 88 implants						Overall *n* = 44 implants					
63.6%	100.0%	100.0%	54.6%	55.6%	A: 1.00 B: 1.00 C: 1.00 D: 1.00 E: 1.00 F: 0.97	47.7%	33.3%	100.0%	53.9%	30.8%	A: 1.00 B: 1.00 C: 0.97 D: 1.00 E: 1.00 F: 0.72
35.2%	33.3%	54.6%	36.4%	25.9%	A: 0.75 B: 0.96 C: 1.00 D: 0.84 E: 0.52 F: 0.86	38.6%	0.0%	0.0%	46.2%	38.5%	A: 1.00 B: 1.00 C: 1.00 D: 1.00 E: 1.00 F: 0.99
9.1%	0.0%	9.1%	13.6%	3.7%	A: 1.00 B: 1.00 C: 1.00 D: 0.98 E: 0.96 F: 0.75	13.6%	0.0%	0.0%	19.2%	7.7%	A: 1.00 B: 1.00 C: 1.00 D: 1.00 E: 1.00 F: 0.97
3.80 ± 1.43	4.47 ± 2.07	3.52 ± 1.06	3.85 ± 1.25	3.69 ± 1.70	A: 0.95 B: 0.95 C: 0.95 D: 0.91 E: 1.00 F: 0.69	3.73 ± 1.49	3.22 ± 0.10	3.67 ± 0.47	3.99 ± 1.34	3.35 ± 1.96	A: 0.69 B: 0.70 C: 0.95 D: 0.99 E: 0.95 F: 0.50
5.11 ± 1.90	5.67 ± 2.25	5.09 ± 1.58	5.27 ± 1.73	4.70 ± 2.25	A: 0.92 B: 0.92 C: 0.74 D: 0.92 E: 0.72 F: 0.58	4.91 ± 1.83	4.33 ± 0.58	5.00 ± 1.41	5.19 ± 1.72	4.46 ± 2.26	A: 0.92 B: 0.88 C: 0.92 D: 0.92 E: 0.92 F: 0.71
0.81 ± 1.05	1.24 ± 1.15	1.33 ± 1.39	0.73 ± 1.06	0.66 ± 0.82	A: 1.00 B: 0.28 C: 0.28 D: 0.28 E: 0.27 F: 0.95	0.47 ± 0.79	0.00 ± 0.00	0.33 ± 0.47	0.31 ± 0.46	0.91 ± 1.21	A: 0.53 B: 0.27 C: 0.27 D: 0.95 E: 0.76 F: 0.28
2.55 ± 1.69	1.17 ± 1.49	1.79 ± 1.49	2.69 ± 1.88	2.93 ± 1.28	A: 0.64 B: 0.25 C: 0.13 D: 0.35 E: 0.15 F: 0.35	2.95 ± 1.72	2.44 ± 1.90	3.67 ± 0.00	3.06 ± 1.93	2.72 ± 1.40	A: 0.82 B: 0.81 C: 1.00 D: 0.63 E: 0.35 F: 0.88

*Note*: A: OFD vs. Impl, B: OFD vs. Rec, C: OFD vs. Comb, D: Impl vs. Rec, E: Impl vs. Comb, F: Rec vs. Comb.

Abbreviation: *n*, number of implants in situ.

**TABLE 6:** Changes in clinical parameters from baseline (T0), to 6 (T1) months, 1 year (T2), 2 years (T3), 3 years (T4), 4 years (T5) and 5 years (T6).

	Baseline‐ T1	OFD *n* = 29 implants	Impl *n* = 26 implants	Rec *n* = 193 implants	Comb *n* = 71 implants	*p*	Baseline‐ T2	OFD *n* = 22 implants	Impl *n* = 24 implants
	Overall *n* = 319 implants						Overall *n* = 263 implants		
PI, %	−12.2%	−3.8%	−5.1%	−13.3%	−14.7%	A: 1.00 B: 1.00 C: 0.99 D: 1.00 E: 0.99 F: 0.78	−11.2	5.9%	7.7%
BOP, %	−57.0%	−54.0%	−44.9%	−63.3%	−45.7%	A: 1.00 B: 0.93 C: 1.00 D: 0.73 E: 0.99 F: 0.68	−58.5%	−69.2%	−49.7%
Sup, %	−36.4%	−43.2%	−23.1%	−37.0%	−38.0%	A: 1.00 B: 1.00 C: 1.00 D: 1.00 E: 1.00 F: 1.00	−38.0%	−43.2%	−18.9%
Mean PD, mm	−1.61 ± 1.36	−1.46 ± 1.65	−1.45 ± 0.83	−1.68 ± 1.41	−1.59 ± 1.22	A: 0.50 B: 1.00 C: 0.87 D: 0.33 E: 0.60 F: 0.56	−1.67 ± 1.28	−1.66 ± 1.69	−0.94 ± 1.88
Max PD, mm	−2.22 ± 1.89	−2.02 ± 1.68	−2.04 ± 1.11	−2.25 ± 2.01	−2.33 ± 1.85	A: 0.61 B: 0.81 C: 0.81 D: 0.61 E: 0.61 F: 0.81	−2.31 ± 1.71	−2.50 ± 1.94	−1.29 ± 2.27
MR, mm	0.25 ± 0.91	−0.02 ± 0.81	0.48 ± 1.24	0.21 ± 0.81	0.42 ± 0.91	A: 0.67 B: 0.99 C: 0.83 D: 0.59 E: 0.87 F: 0.59	0.24 ± 0.88	0.25 ± 0.88	0.28 ± 0.95
KM, mm	+0.14 ± 1.62	+0.32 ± 1.64	+0.16 ± 1.71	+0.19 ± 1.58	−0.16 ± 1.52	A: 0.90 B: 0.90 C: 0.90 D: 1.00 E: 0.90 F: 0.90	0.12 ± 1.52	0.07 ± 1.62	0.73 ± 1.71

	Rec *n* = 159 implants	Comb *n* = 58 implants	*p*	Baseline‐ T3	OFD *n* = 11 implants	Impl *n* = 14 implants	Rec *n* = 110 implants	Comb *n* = 48 implants	*p*
				Overall *n* = 183 implants					
PI, %	−10.6%	−27.0%	A: 1.00 B: 1.00 C: 1.00 D: 1.00 E: 0.92 F: 0.52	−3.2%	15.0%	−1.8%	2.6%	−22.8%	A: 1.00 B: 1.00 C: 1.00 D: 1.00 E: 1.00 F: 0.99
BOP, %	−58.6%	−58.0%	A: 1.00 B: 0.95 C: 1.00 D: 0.78 E: 1.00 F: 0.61	−54.9%	−91.9%	−15.8%	−58.6%	−49.5%	A: 1.00 B: 1.00 C: 1.00 D: 0.11 E: 0.11 F: 1.00
Sup, %	−38.1%	−43.7%	A: 1.00 B: 1.00 C: 1.00 D: 1.00 E: 1.00 F: 1.00	−33.2%	−43.2%	−15.9%	−37.0%	−28.5%	A: 1.00 B: 1.00 C: 1.00 D: 1.00 E: 1.00 F: 0.80
Mean PD, mm	−1.78 ± 1.02	−1.70 ± 1.46	A: 0.49 B: 0.98 C: 0.70 D: 0.46 E: 0.52 F: 0.87	−1.40 ± 1.39	−1.24 ± 1.44	−0.82 ± 1.36	−1.54 ± 1.27	−1.40 ± 1.68	A: 0.87 B: 0.98 C: 0.97 D: 0.31 E: 0.31 F: 0.99
Max PD, mm	−2.43 ± 1.45	−2.36 ± 2.02	A: 0.61 B: 0.81 C: 0.81 D: 0.61 E: 0.61 F: 0.89	−1.87 ± 1.85	−1.90 ± 1.96	−1.64 ± 1.65	−1.94 ± 1.73	−1.90 ± 2.14	A: 1.00 B: 0.87 C: 0.73 D: 0.70 E: 0.61 F: 0.73
MR, mm	0.20 ± 0.77	0.36 ± 0.98	A: 0.87 B: 0.43 C: 0.67 D: 0.43 E: 0.87 F: 0.43	0.36 ± 1.00	0.00 ± 0.88	0.95 ± 1.53	0.29 ± 0.81	0.51 ± 1.03	A: 0.88 B: 0.59 C: 0.69 D: 0.59 E: 0.59 F: 0.83
KM, mm	0.10 ± 1.44	−0.13 ± 1.57	A: 0.90 B: 0.90 C: 0.90 D: 0.90 E: 0.90 F: 0.90	0.42 ± 1.56	0.94 ± 1.80	1.02 ± 1.86	0.35 ± 1.55	0.17 ± 1.42	A: ‐ B: ‐ C: ‐ D: 0.70 E: 0.70 F: 0.70

Baseline‐ T4	OFD *n* = 8 implants	Impl *n* = 11 implants	Rec *n* = 85 implants	Comb *n* = 32 implants	*p*	Baseline‐ T5	OFD *n* = 6 implants	Impl *n* = 11 implants	Rec *n* = 44 implants	Comb *n* = 28 implants	*p*
Overall *n* = 136 implants						Overall *n* = 89 implants					
−9.3%	1.4%	−4.4%	−5.3%	−24.3%	A: 1.00 B: 1.00 C: 1.00 D: 1.00 E: 1.00 F: 1.00	12.9%	51.4%	41.0%	8.1%	−4.2%	A: ‐ B: ‐ C: ‐ D: 1.00 E: 1.00 F: 0.73
−49.4%	−54.4%	−59.9%	−50.1%	−42.4%	A: 1.00 B: 1.00 C: 1.00 D: 0.96 E: 0.72 F: 0.73	−55.6%	−58.6%	−32.6%	−54.9%	−64.9%	A: ‐ B: ‐ C: ‐ D: 0.57 E: 0.49 F: 0.96
−32.4%	−43.2%	−23.1%	−30.1%	−40.5%	A: 1.00 B: 1.00 C: 1.00 D: 1.00 E: 1.00 F: 1.00	−30.8%	−43.2%	−14.0%	−27.0%	−40.0%	A: ‐ B: ‐ C: ‐ D: 0.92 E: 0.96 F: 0.92
−1.37 ± 1.27	−0.99 ± 1.97	−0.99 ± 0.97	−1.40 ± 1.28	−1.63 ± 1.12	A: 0.87 B: 1.00 C: 1.00 D: 0.52 E: 0.52 F: 1.00	−1.26 ± 1.43	−0.27 ± 2.07	−0.87 ± 1.06	−1.35 ± 1.25	−1.42 ± 1.70	—
−1.81 ± 1.70	−1.42 ± 2.53	−1.45 ± 1.44	−1.70 ± 1.68	−2.45 ± 1.52	A: 0.61 B: 0.61 C: 0.81 D: 0.73 E: 0.61 F: 0.61	−1.72 ± 1.91	−0.87 ± 2.25	−0.91 ± 1.58	−1.67 ± 1.73	−2.26 ± 2.25	—
0.29 ± 0.94	0.46 ± 1.19	0.54 ± 1.18	0.24 ± 0.80	0.39 ± 0.95	A: 1.00 B: 0.99 C: 0.87 D: 0.87 E: 0.67 F: 0.59	0.41 ± 1.06	0.34 ± 1.15	0.50 ± 1.39	0.47 ± 1.06	0.23 ± 0.82	—
0.54 ± 1.73	0.17 ± 1.76	0.33 ± 2.01	0.63 ± 1.66	0.32 ± 1.60	A: 1.00 B: 1.00 C: 1.00 D: 1.00 E: 1.00 F: 1.00	0.01 ± 1.70	−1.08 ± 1.49	0.33 ± 1.49	−0.06 ± 1.88	0.34 ± 1.28	—

Baseline ‐ T6	OFD Note; *n* = 3 implants	Impl *n* = 2 implants	Rec *n* = 26 implants	Comb *n* = 13 implants	*p*
Overall *n* = 44 implants					
−3.0%	−15.3%	41.0%	7.4%	−29.0%	A: 1.00 B: 1.00 C: 1.00 D: 1.00 E: 1.00 F: 0.98
−52.2%	−91.9%	−87.2%	−45.1%	−52.3%	A: 1.00 B: 1.00 C: 1.00 D: 1.00 E: 1.00 F: 0.42
−26.2%	−43.2%	−23.1%	−21.4%	−36.0%	A: 1.00 B: 1.00 C: 1.00 D: 1.00 E: 1.00 F: 0.95
−1.32 ± 1.49	−1.52 ± 0.10	−0.72 ± 0.47	−1.20 ± 1.34	−1.76 ± 1.96	—
−1.91 ± 1.83	−2.21 ± 0.58	−1.00 ± 1.41	−1.75 ± 1.72	−2.50 ± 2.26	—
0.06 ± 0.79	−0.90 ± 0.00	−0.49 ± 0.47	0.05 ± 0.46	0.48 ± 1.21	—
0.41 ± 1.72	0.20 ± 1.90	2.21 ± 0.00	0.32 ± 1.93	0.14 ± 1.40	—

*Note*: −: Due to limited data, regression was not performed for the T5 and T6 time points. A: OFD vs. Impl, B: OFD vs. Rec, C: OFD vs. Comb, D: Impl vs. Rec, E: Impl vs. Comb, F: Rec ve. Comb.

Abbreviation: *n*, number of implants in situ.

### 3.5 Regression Analyses

For treatment success at T2 (1 year), the use of systemic antibiotics and the surgeon‐related differences emerged as independent predictors. Implants treated with systemic antibiotics—whether administered pre‐operatively or both pre‐ and post‐operatively—showed a higher probability of success. Treatment success differed among surgeons, with one surgeon showing significantly lower success compared with the reference surgeon.

For implant loss, significant independent predictors included implant location, antibiotic regimen and the presence of Sup at baseline. Implants placed in the molar region and those presenting with baseline suppuration showed a higher likelihood of implant loss during follow‐up. In addition, pre‐ and post‐operative systemic antibiotic use was associated with an increased risk of implant loss.

For the need for surgical retreatment, prosthesis type and baseline max PD were identified as independent predictors. Overdenture prostheses and deeper baseline PDs were associated with higher odds of requiring retreatment. Each 1 mm increase in baseline max PD increased the odds by about 30%.

All independent predictors and their corresponding adjusted OR, 95% CI and *p*‐values are summarised in Table 7.

**TABLE 7:** Independent predictors of treatment success, implant loss and surgical retreatment identified in multivariable mixed‐effects logistic regression models.

Outcome	Independent predictor	Adjusted OR	95% CI	*p*
Treatment success (T2, Criterion 1)	Pre‐operative systemic antibiotics (vs. no antibiotics)	3.54	1.09–11.50	0.04*
	Post‐operative systemic antibiotics (vs. no antibiotics)	2.15	0.58–7.94	0.25
	Pre‐ + post‐operative systemic antibiotics (vs. no antibiotics)	4.49	1.24–16.28	0.02*
	Surgeon 1 (vs. most experienced)	0.68	0.21–2.15	0.51
	Surgeon 2 (vs. most experienced)	0.12	0.03–0.50	0.003*
	Surgeon 3 (vs. most experienced)	1.16	0.32–4.13	0.82
	Max PD (per mm increase)	0.55	0.27–1.12	0.10
	Mean PD (per mm increase)	0.91	0.44–1.85	0.79
Implant loss	Diabetes (vs. no general diseases)	0.70	0.02–20.08	0.83
	Cardiovascular (vs. no general diseases)	2.50	0.18–5.28	0.15
	Osteoporosis (vs. no general diseases)	0.99	0.16–5.91	0.99
	Combined (vs. no general diseases)	0.94	0.21–4.01	0.93
	Premolar site (vs. anterior)	1.95	0.63–6.04	0.25
	Molar site (vs. anterior)	3.21	1.11–9.25	0.03*
	Pre‐operative systemic antibiotics (vs. no antibiotics)	1.78	0.70–6.04	0.23
	Post‐operative systemic antibiotics (vs. no antibiotics)	2.35	0.85–6.50	0.10
	Pre‐ + post‐operative systemic antibiotics (vs. no antibiotics)	3.13	1.20–8.17	0.02*
	Suppuration at baseline (per % increase)	4.51	1.74–11.66	0.002*
Surgical retreatment	Light smoker (< 10 cig./day) (vs. non‐smoker)	2.45	0.78–7.65	0.12
	Heavy smoker (≥ 10 cig./day) (vs. non‐smoker)	1.51	0.52–4.40	0.44
	Bridge (vs. single crown)	2.67	0.82–1.87	0.28
	Full‐arch fixed prosthesis (vs. single crown)	2.57	0.60–11.03	0.20
	Overdenture prosthesis (vs. single crown)	3.59	1.54–8.36	0.003*
	Max PD (per mm increase)	1.30	1.11–1.52	0.001*

* Indicates the difference is statistically significant.

## 4 Discussion

This retrospective cohort study evaluated the treatment outcome of surgical peri‐implantitis treatment protocols performed by trained surgeons at a single university centre, thereby minimising confounding factors as much as possible. Using consensus‐based success Criterion 1 (Berglundh, Armitage, et al. 2018), 54.7% of implants were successfully treated at 1 year, and 26.9% of implants after 5 years, indicating some decline over time and overall maintenance of peri‐implant health in more than one‐fourth of the cases. No significant differences in success rates were observed between different treatment modalities at any time point. These results fall within the range of treatment success reported in previous clinical studies applying the same or similar success criteria. A university‐based study by two experienced clinicians reported reconstructive peri‐implantitis treatment success of 41.7%–50% at 1 year, dropping to 25%–35.3% at 7 years (Isler et al. 2025). A multi‐centre study (universities and private practices) found disease resolution after 3 years in 46.3% of implants with non‐reconstructive treatment and 30% with reconstructive treatment, with no significant difference (Alibegovic et al. 2025), although confounding factors were noted due to different clinicians. And a single‐surgeon case series showed reconstructive therapy success declining from 91% at 1 year to 79% at 3 years and 59% at 5 years (La Monaca et al. 2018).

The definition of ‘success’ strongly influences the reported outcomes. The ≤ 5 mm PD threshold is widely recommended but may be clinically misleading in cases with deep insertion or thick soft tissue, where 6–7 mm PDs can exist without disease (Monje et al. 2018; Serino et al. 2013; García‐García et al. 2021; Fuchigami et al. 2017). Therefore, criteria 2 and 3 were introduced as complementary improvement‐based definitions to capture clinically meaningful reductions in PD even in cases with residual deep pockets. These criteria provide a more realistic reflection of treatment response while maintaining clinical relevance. Importantly, the outcome variations between criteria 1 and 3 were mainly driven by the slightly more permissive BOP threshold (≤ 2 sites vs. ≤ 1), underscoring that bleeding tendency rather than PD largely accounted for outcome variability. When interpreting success rates across the four modalities, limited sample sizes (particularly in OFD and Impl), group imbalance and heterogeneity in defect morphology reduce effective statistical power. Therefore, comparisons—especially Impl versus OFD—should be interpreted as exploratory.

In the present study, over a 5‐year follow‐up period, a total of 40 implants (9.9%) were lost in 30 patients (12.9%). These rates are lower than those previously reported following non‐reconstructive surgical peri‐implantitis therapy, where implant loss occurred in 19.9% (53/267) of the cases after 4.4 years (Romandini et al. 2024). In a long‐term study of reconstructive peri‐implantitis surgery in 26 patients, no implant loss occurred at 1 year, while failure rates increased to 15% at 7 years and 27% at 10 years (Roccuzzo et al. 2020). Surgical retreatment was performed for 38 implants (9.4%) across 22 patients. This rate is lower than that reported by Romandini et al. (24.2% over 7 years after non‐reconstructive therapy) and other reconstructive studies (18%–31% retreatment over 7–10 years) (Isler et al. 2025; Roccuzzo et al. 2020). These variations may reflect non‐standardised retreatment criteria inherent to retrospective designs.

Upon further analysis of the present dataset, systemic antibiotic use was associated with higher odds of treatment success at 1 year (pre‐operative antibiotics: OR = 3.54, *p* = 0.04; pre‐ and post‐operative antibiotics: OR = 4.49, *p* = 0.02). However, pre‐ and post‐operative antibiotic use was also associated with an increased risk of implant loss over the longer follow‐up period (OR = 3.13, *p* = 0.02). The apparent discrepancy between higher short‐term treatment success and increased long‐term implant loss likely reflects confounding by indication and the time‐dependent nature of treatment outcomes. Systemic antibiotics were preferentially prescribed in more severe cases, which may achieve short‐term clinical resolution following surgery, yet remain biologically compromised and at higher risk for late implant loss. The observed benefits following antibiotics use in the present study contrast with previous a randomised clinical study, which failed to identify any clinical benefit of adjunctive systemic antibiotics prescribed following non‐reconstructive surgical treatment on clinical outcomes (BOP, PD and Sup reductions) at 12 months (Hallström et al. 2017). Other randomised controlled trials, however, reported significantly higher treatment success (PD ≤ 5 mm, no BOP/Sup and no bone loss > 0.5 mm) at 12 months for modified‐surface implants treated with a non‐reconstructive/resective approach combined with adjunctive systemic antibiotics (Carcuac et al. 2016; Riben Grundström et al. 2024). However, the observed benefit of systemic antibiotics was not sustained over a 3‐year follow‐up period (Carcuac et al. 2017). Taken together, these findings suggest that systemic antibiotics may be associated with improved short‐term clinical endpoints without translating into improved long‐term implant survival, supporting current S3 clinical practice guidelines that advise against the routine use of systemic antibiotics as an adjunct to surgical peri‐implantitis therapy (Herrera et al. 2023).

This study was conducted at a single university centre with four trained surgeons using predefined treatment protocols and consistent decision‐making for treatment selection. Despite this, significant differences in treatment success were observed among surgeons, partially aligning with a retrospective study highlighting the surgeon‐related differences as a key factor in resective peri‐implantitis therapy outcomes (de Waal et al. 2016).

The presence of Sup at baseline increased the odds of implant loss by more than 4.5‐fold, aligning with a retrospective study that identified baseline Sup as a significant predictor of implant loss over 7 years following non‐reconstructive therapy (Romandini et al. 2024). Moreover, the risk of implant failure after surgical treatment for peri‐implantitis is approximately 3.2 times higher in molar sites than in anterior sites, likely because molars are harder to debride and maintain, have poorer oral hygiene access and experience greater occlusal loading—factors that may promote bone resorption and compromise implant stability (Schwarz et al. 2018).

Implants supporting overdentures had 3.6‐fold higher odds of requiring surgical retreatment than those with single crowns, possibly due to challenges in plaque control with removable prostheses (Grischke et al. 2021). Furthermore, baseline max PD values were positively associated with the need for surgical retreatment, consistent with previous studies identifying baseline PD as a key predictor of clinical outcomes following peri‐implantitis therapies, including final PD, recession, marginal bone gain and pocket closure (PD < 5 mm) (Ichioka et al. 2023; Koldsland et al. 2018; Isler et al. 2025).

This retrospective study, despite unified treatment protocols, has several limitations. The retrospective design precluded an a priori sample size calculation. In combination with small and uneven group sizes—particularly in the Impl and OFD groups—and loss to follow‐up, the study had limited statistical power for the primary 1‐year analyses. Consequently, the absence of statistically significant differences between treatment approaches should be interpreted with caution, as the study was likely underpowered to detect small to moderate effects. Furthermore, assessment of peri‐implant clinical parameters was not always performed at fixed 6‐ or 12‐month intervals; only implants with available data at each predefined timepoint were included in the analyses, which may have affected the comparability across follow‐up intervals. Moreover, non‐randomised treatment allocation based on defect configuration and patient‐specific considerations introduced selection bias and confounding, which may have influenced the observed associations despite the mixed‐effects model accounting for intra‐patient clustering. Additionally, due to the retrospective nature of data collection, detailed information regarding the defect configuration (i.e., number of remaining bone walls, extent of the defect, etc.) could not be assessed. This is particularly relevant, as previous studies have demonstrated that the outcomes of reconstructive peri‐implantitis therapy are closely associated with the defect type, with four‐wall defects displaying 6.0 to 7.0 times higher odds of successful outcomes compared to three‐ or two‐wall defects (Isler et al. 2022). From a statistical perspective, given the limited number of events, the multivariable models approached the lower bound of acceptable events per variable (EPV) ratios. As a sensitivity analysis, the Firth logistic regression was performed (without random effects), yielding consistent results. All multivariable findings should be interpreted as exploratory. It should be further acknowledged that according to the current S3 clinical practice guideline, treatment success should also include the absence of progressive bone loss on follow‐up radiographs (Herrera et al. 2023). In this retrospective dataset, standardised radiographic follow‐up was unavailable for many cases; thus, success was defined clinically. To account for this limitation, complementary criteria 2 and 3 were introduced to reflect clinically meaningful improvements in PD and BOP. Furthermore, the choice of perioperative systemic antibiotic regimen was not based on predefined criteria, introducing potential heterogeneity and representing an additional limitation. Finally, assessment of the clinical measurements at baseline and follow‐up appointments by treating surgeons might have introduced detection bias, potentially influencing the assessed clinical outcomes.

## 5 Conclusions

Within the limitations of this retrospective exploratory analysis, surgical therapy for peri‐implantitis achieved a 1‐year success rate of 54.7%, with no significant differences observed among surgical modalities. Treatment success was associated with systemic antibiotic use (pre‐ or pre‐ and post‐operative) and surgeon‐related differences. In contrast, implant loss was associated with molar sites, baseline suppuration and pre‐ and post‐operative antibiotics. Surgical retreatment was linked to overdentures and greater baseline PD. These findings highlight the complex influence of case selection, operator factors and antibiotic prescribing patterns on outcomes.

## Author Contributions

A.R. and F.S. conceived the idea and designed the study. W.Z., C.R. and N.L. acquired and analysed the data. A.R., F.S. and E.A.C. interpreted the data. A.R. and W.Z. led the writing. F.S. critically revised the manuscript. All authors gave final approval and agreed to be accountable for all aspects of the scientific work.

## Funding

The study was funded by the Department of Oral Surgery, Implantology and Oral Medicine, Goethe University, Carolinum, Frankfurt am Main, Germany.

## Ethics Statement

The study protocol was approved by the ethics committee of Goethe University Frankfurt, Carolinum (registration number: 2025‐2337).

## Consent

Written informed consent was obtained from all patients included in this study.

## Conflicts of Interest

Frank Schwarz, Ausra Ramanauskaite and Emilio A. Cafferata have received research grants and lecture fees from Geistlich Pharma AG and the Osteology Foundation (OF). Frank Schwarz is the president of the OF and Ausra Ramanauskaite is Expert Council member of the OF. Wenjie Zhou and Emilio A. Cafferata have received Scholarship Grants from the OF. Clemens Raabe and Neelam Lingwal have no conflicts of interest to report pertaining to this study.

## Supporting information


**Figure S1:** Overall PI, BOP and Sup at baseline and during the follow‐up visits.

**Figure S2:** Overall mean PD, max PD, MR and KM at baseline and during the follow‐up visits.

**Table S1:** Preoperative treatment and systemic antibiotic regimen
**Table S2a:** Treatment success—Criterion 1.
**Table S2b:** Treatment success—Criterion 2.
**Table S2c:** Treatment success—Criterion 3.
**Table S3:** Regression analysis using a univariate regression model: Influence of different patient‐ and implant‐related factors and treatment modalities on treatment outcomes.

## Data Availability

The data that support the findings of this study are available on request from the corresponding author. The data are not publicly available due to privacy or ethical restrictions.
