# Supplementary figures and images for: Treatment Outcome of Surgical Protocols for Peri‐Implantitis: A Retrospective Cohort Study in a Specialised University Centre
Source: J Clin Periodontol. 2026 Mar 15;53(5):658–80. doi: 10.1111/jcpe.70115 (PMC13086545; doi:10.1111/jcpe.70115)

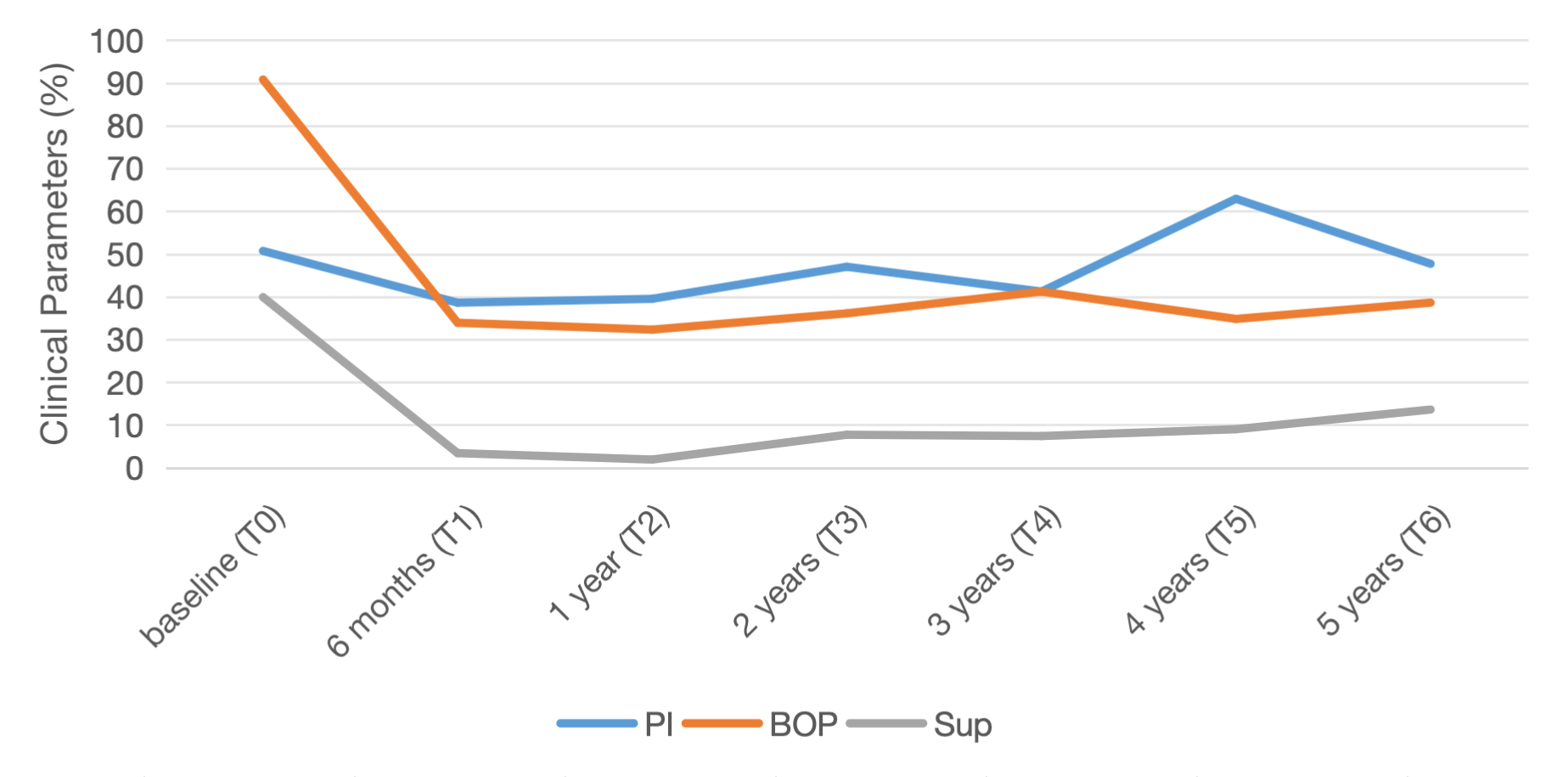

Supplement: Supplementary file 1 — Figure S1: Overall PI, BOP and Sup at baseline and during the follow‐up visits. [file JCPE-53-658-s002.png]

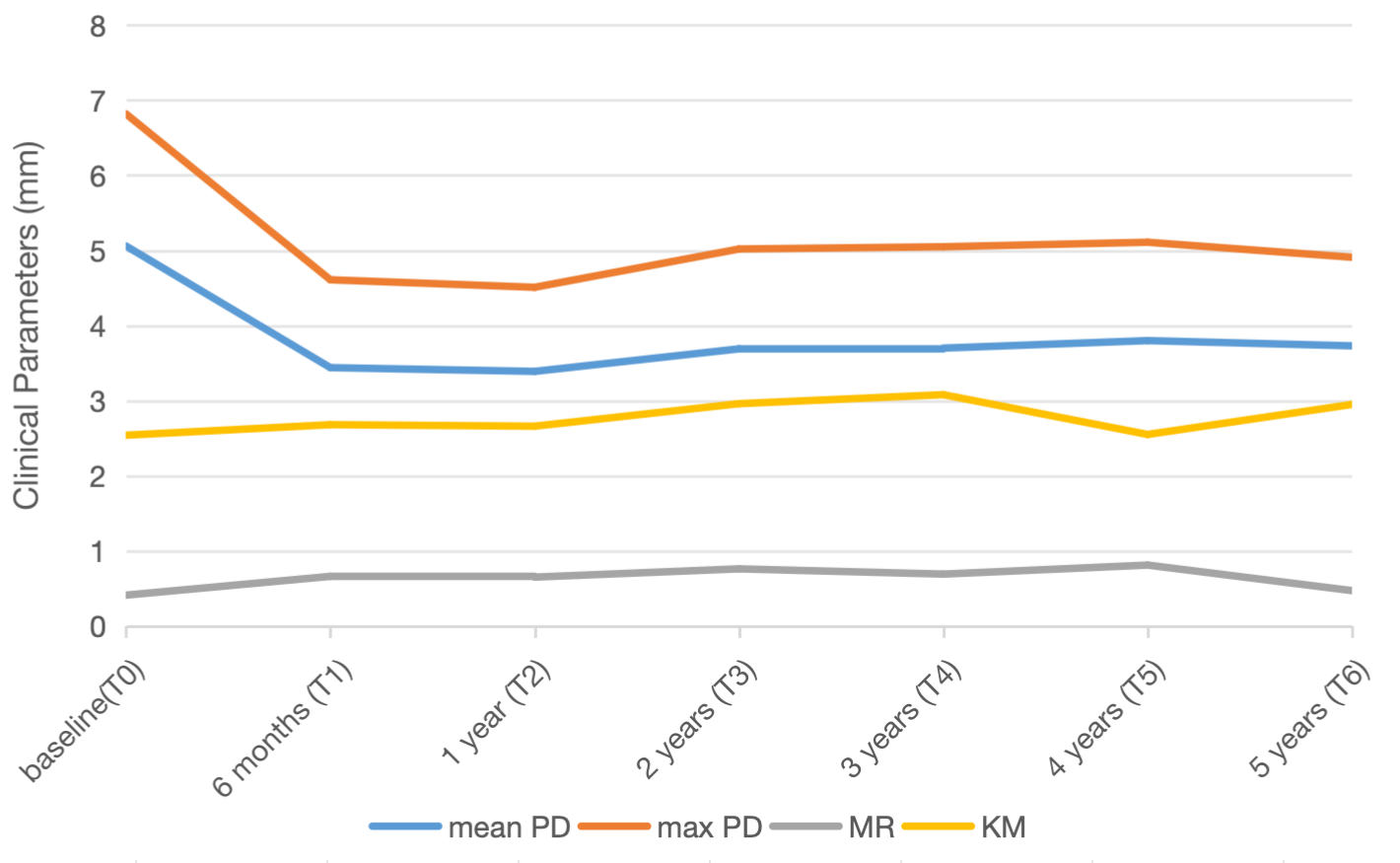

Supplement: Supplementary file 2 — Figure S2: Overall mean PD, max PD, MR and KM at baseline and during the follow‐up visits. [file JCPE-53-658-s001.png]
