# Supplementary material for: Treatment Outcome of Surgical Protocols for Peri‐Implantitis: A Retrospective Cohort Study in a Specialised University Centre
Source: J Clin Periodontol. 2026 Mar 15;53(5):658–80. doi: 10.1111/jcpe.70115 (PMC13086545; doi:10.1111/jcpe.70115)
Supplement: Supplementary file 3 — Table S1: Preoperative treatment and systemic antibiotic regimen Table S2a: Treatment success—Criterion 1. Table S2b: Treatment success—Criterion 2. Table S2c: Treatment success—Criterion 3. Table S3: Regression analysis using a univariate regression model: Influence of different patient‐ and implant‐related factors and treatment modalities on treatment outcomes. [file JCPE-53-658-s003.docx]

Table S1. Preoperative treatment and systemic antibiotic regimen

| Preoperative treatment | Patients subjected to preoperative treatment 2 to 6 weeks prior to surgery that included professional supramucosal/gingival implant/tooth cleaning along with a single session of non-surgical therapy employing any of the following measures: |
| --- | --- |
|  | - Er:YAG laser (KEY3; KaVo, Biberach, Germany) (12.7 J/cm^2^); |
|  | - Air-flow device with glycine powder (EMS® Airflow), |
|  | - Chitosan brush (Labrida BioClean, Labrida AS, Oslo, Norway); |
|  | - Mechanical debridement with titanium curettes and chlorhexidine irrigation (CHX 0.12%). |
|  | Patients not receiving preoperative treatment. |
| Systemic antibiotic regimen | Pre-op-Ab: Patients who received a single preoperative oral dose of antibiotics (2 g amoxicillin; Amoxicillin, Aliud Pharma, Laichingen, Germany). In cases of allergy, clindamycin 600 mg (Clindamycin-ratiopharm, Merckle GmbH, Blaubeuren, Germany) was administered instead. |
|  | Post-op-Ab: Patients who received postoperative antibiotics for 5 days (500 mg amoxicillin, three times daily; Amoxicillin, Aliud Pharma, Laichingen, Germany). In cases of allergy, clindamycin 300 mg (Clindamycin-ratiopharm, Merckle GmbH, Blaubeuren, Germany) was prescribed. |
|  | Pre+post-op-Ab: Patients who received both a single preoperative oral dose (2 g amoxicillin) and postoperative antibiotics for 5 to 7 days. In cases of allergy, clindamycin 300 mg (Clindamycin-ratiopharm, Merckle GmbH, Blaubeuren, Germany) was used. |
|  | No-Ab: Patients who did not receive any systemic antibiotics. |

**TABLE S2a.** Treatment success – Criteria 1.

|  | **Overall**  **(%)** | **OFD (%)** | **Impl (%)** | **Rec (%)** | **Comb (%)** | ***p* value** |
| --- | --- | --- | --- | --- | --- | --- |
| 6 mo (T1)  n/T | 59.1%  192/325 | 62.1%  18/29 | 57.7%  15/26 | 62.3%  124/199 | 49.3%  35/71 | A: 0.89  B: 0.98  C: 0.74  D: 0.89  E: 0.89  F: 0.34 |
| 1 year (T2)  n/T | 54.7%  150/274 | 62.5%  15/24 | 58.3%  14/24 | 55.4%  92/166 | 48.3%  29/60 | A: 0.79  B: 0.77  C: 0.77  D: 0.79  E: 0.77  F: 0.77 |
| 2 year (T3)  n/T | 47.8%  96/201 | 64.3%  9/14 | 26.7%  4/15 | 51.7%  62/120 | 40.4%  21/52 | A: 0.23  B: 0.38  C: 0.24  D: 0.24  E: 0.38  F: 0.18 |
| 3 year (T4)  n/T | 31.9%  52/163 | 27.3%  3/11 | 46.2%  6/13 | 29.6%  29/98 | 34.1%  14/41 | A: 0.80  B: 0.87  C: 0.80  D: 0.80  E: 0.80  F: 0.80 |
| 4 year (T5)  n/T | 38.0%  46/121 | 33.3%  3/9 | 35.7%  5/14 | 34.5%  20/58 | 45.0%  18/40 | A: 0.95  B: 0.95  C: 0.95  D: 0.95  E: 0.95  F: 0.95 |
| 5 year (T6)  n/T | 26.9%  21/78 | 50.0%  3/6 | 20.0%  1/5 | 24.4%  10/41 | 26.9%  7/26 | A: 0.63  B: 0.63  C: 0.63  D: 0.83  E: 0.83  F: 0.83 |

Treatment success defined as max PD ≤ 5 mm, BOP ≤ 1 site, no Sup; n/T: successfully treated implants/total treated implants; A: OFD vs. Impl, B: OFD vs. Rec, C: OFD vs. Comb, D: Impl vs. Rec, E: Impl vs. Comb, F:Rec vs. Comb.

**TABLE S2b.** Treatment success – Criteria 2.

|  | **Overall**  **(%)** | **OFD (%)** | **Impl (%)** | **Rec (%)** | **Comb (%)** | ***p* value** |
| --- | --- | --- | --- | --- | --- | --- |
| 6 mo (T1)  n/T | 62.5%  203/325 | 58.6%  17/29 | 53.8%  14/26 | 67.8%  135/199 | 52.1%  37/71 | A: 0.87  B: 0.66  C: 0.83  D: 0.48  E: 0.88  F: 0.11 |
| 1 year (T2)  n/T | 63.1%  173/274 | 70.8%  17/24 | 58.3%  14/24 | 62.0%  103/166 | 65.0%  39/60 | A: 0.73  B: 0.73  C: 0.73  D: 0.73  E: 0.73  F: 0.73 |
| 2 year (T3)  n/T | 53.2%  107/201 | 78.6%  11/14 | 20.0%  3/15 | 58.3%  70/120 | 44.2%  23/52 | A: 0.02*  B: 0.16  C: 0.06  D: 0.03*  E: 0.12  F: 0.12 |
| 3 year (T4)  n/T | 41.7%  68/163 | 45.5%  5/11 | 53.8%  7/13 | 42.9%  42/98 | 34.1%  14/41 | A: 0.82  B: 0.87  C: 0.74  D: 0.74  E: 0.74  F: 0.74 |
| 4 year (T5)  n/T | 42.1%  51/121 | 44.4%  4/9 | 28.6%  4/14 | 41.4%  24/58 | 47.5%  19/40 | A: 0.82  B: 0.87  C: 0.87  D: 0.82  E: 0.82  F: 0.82 |
| 5 year (T6)  n/T | 29.5%  23/78 | 50.0%  3/6 | 40.0%  2/5 | 26.8%  11/41 | 26.9%  7/26 | A: 0.89  B: 0.84  C: 0.84  D: 0.84  E: 0.84  F: 0.99 |

Treatment success defined as reduction in max PD, BOP ≤ 2 site, no Sup; n/T: successfully treated implantsimplants/total treated implants. A: OFD vs. Impl, B: OFD vs. Rec, C: OFD vs. Comb, D: Impl vs. Rec, E: Impl vs. Comb, F:Rec vs. Comb.

**TABLE S2c.** Treatment success – Criteria 3.

|  | **Overall (%)** | **OFD (%)** | **Impl (%)** | **Rec (%)** | **Comb (%)** | ***p* value** |
| --- | --- | --- | --- | --- | --- | --- |
| 6 mo (T1)  n/T | 57.5%  187/325 | 55.2%  16/29 | 53.8%  14/26 | 62.8%  125/199 | 45.1%  32/71 | A: 0.92  B: 0.53  C: 0.53  D: 0.53  E: 0.53  F: 0.06 |
| 1 year (T2)  n/T | 55.8%  153/274 | 58.3%  14/24 | 41.7%  10/24 | 56.0%  93/166 | 60.0%  36/60 | A: 0.50  B: 0.89  C: 0.89  D: 0.50  E: 0.50  F: 0.89 |
| 2 year (T3)  n/T | 48.3%  97/201 | 71.4%  10/14 | 26.7%  4/15 | 50.0%  60/120 | 44.2%  23/52 | A: 0.12  B: 0.21  C: 0.20  D: 0.20  E: 0.27  F: 0.49 |
| 3 year (T4)  n/T | 39.3%  64/163 | 36.4%  4/11 | 61.5%  8/13 | 39.8%  39/98 | 31.7%  13/41 | A: 0.45  B: 0.83  C: 0.83  D: 0.44  E: 0.37  F: 0.56 |
| 4 year (T5)  n/T | 38.8%  47/121 | 44.4%  4/9 | 35.7%  5/14 | 32.8%  19/58 | 47.5%  19/40 | A: 0.87  B: 0.87  C: 0.87  D: 0.87  E: 0.87  F: 0.86 |
| 5 year (T6)  n/T | 28.2%  22/78 | 50.0%  3/6 | 40.0%  2/5 | 22.0%  9/41 | 30.8%  8/26 | A: 0.74  B: 0.63  C: 0.63  D: 0.63  E: 0.74  F: 0.63 |

Treatment success defined as reduction in max PD, BOP ≤ 2 site, no Sup; n/T: successfully treated implants/total treated implants. A: OFD vs. Impl, B: OFD vs. Rec, C: OFD vs. Comb, D: Impl vs. Rec, E: Impl vs. Comb, F:Rec vs. Comb.

**TABLE S3.** Regression analysis using an univariate regression model: Influence of different patient- and implant-related factors and treatment modalities on treatment outcomes.

|  | | | **On overall success (1 year, criteria 1)** | | | **Implant loss** | | | **Surgical retreatment** | | |
| --- | --- | --- | --- | --- | --- | --- | --- | --- | --- | --- | --- |
|  | | | OR | 95% CI | *p* value | OR | 95% CI | *p* value | OR | 95% CI | *p* value |
| Patient related | Age |  | 0.99 | 0.96–1.02 | 0.63 | 1.00 | 0.97–1.03 | 0.88 | 1.01 | 0.98–1.04 | 0.55 |
|  | Gender | Female/Male | 1.16 | 0.55–2.42 | 0.70 | 0.95 | 0.49–1.85 | 0.88 | 1.27 | 0.65–2.46 | 0.49 |
|  | Smoking habit | Non-smoker | Ref. | - | - | Ref. | - | - | Ref. | - | - |
|  |  | Light smoker  (< 10 cig/day) | 0.93 | 0.22–3.95 | 0.93 | 2.14 | 0.88–5.20 | 0.09 | 3.09 | 1.34–7.09 | 0.008* |
|  |  | Heavy smoker (≥ 10 cig/day) | 0.92 | 0.26–3.34 | 0.90 | 1.96 | 0.77–4.98 | 0.16 | 1.74 | 0.64–4.70 | 0.28 |
|  | General diseases | None | Ref. | - | - | Ref. | - | - | Ref. | - | - |
|  |  | Diabetes | 1.21 | 0.06–22.57 | 0.90 | 0 | 0 | 0 | 0 | 0 | 0 |
|  |  | Cardiovascular | 0.97 | 0.41–2.28 | 0.95 | 2.36 | 1.18–4.72 | 0.01* | 0.90 | 0.44–1.87 | 0.78 |
|  |  | Osteoporosis | 3.39 | 0.47–24.58 | 0.22 | 0.82 | 0.14–4.76 | 0.82 | 0.18 | 0.01–3.18 | 0.24 |
|  |  | Combined | 1.59 | 0.32–7.91 | 0.57 | 1.04 | 0.26–4.24 | 0.96 | 1.09 | 0.33–3.67 | 0.89 |
| Implant site | Region | Anterior | Ref. | - | - | Ref. | - | - | Ref. | - | - |
|  |  | Premolar | 1.44 | 0.56–3.72 | 0.45 | 1.58 | 0.52–4.82 | 0.42 | 0.80 | 0.35–1.86 | 0.61 |
|  |  | Molar | 0.71 | 0.28–1.81 | 0.47 | 3.01 | 1.05–8.60 | 0.04* | 0.64 | 0.27–1.52 | 0.32 |
|  | Jaw | Maxilla/Mandible | 1.07 | 0.53–2.14 | 0.86 | 1.84 | 0.96–3.56 | 0.07 | 0.59 | 0.29–1.22 | 0.16 |
|  | Location | Anterior maxilla | Ref. | - | - | Ref. | - | - | Ref. | - | - |
|  |  | Anterior mandible | 0.90 | 0.15–5.27 | 0.90 | 1.47 | 0.19–11.12 | 0.71 | 0.14 | 0.01–2.65 | 0.19 |
|  |  | Posterior maxilla | 0.90 | 0.32–2.52 | 0.85 | 1.76 | 0.53–5.88 | 0.36 | 0.61 | 0.26–1.39 | 0.24 |
|  |  | Posterior mandible | 1.06 | 0.36–3.11 | 0.92 | 2.96 | 0.91–9.66 | 0.07 | 0.46 | 0.19–1.14 | 0.09 |
| Implant and prosthsis type | Implant type | Bone level/Tissue level | 1.12 | 0.38–3.30 | 0.84 | 0.48 | 0.13–1.82 | 0.28 | 0.73 | 0.23–2.30 | 0.59 |
|  | Prosthetic type | Single crown | Ref. | - | - | Ref. | - | - | Ref. | - | - |
|  |  | Bridge | 0.98 | 0.45–2.12 | 0.96 | 1.02 | 0.50 –2.11 | 0.95 | 2.40 | 0.70–7.78 | 0.02* |
|  |  | Full-arch fixed prosthesis | 1.91 | 0.07–49.20 | 0.70 | 1.47 | 0.34–6.41 | 0.61 | 4.61 | 1.43–14.88 | 0.01* |
|  |  | Overdenture | 0.48 | 0.16–1.39 | 0.17 | 0.64 | 0.23–1.74 | 0.38 | 2.39 | 1.11–5.15 | 0.03* |
| Treatment | Antibiotic regimen | None | Ref. | - | - | Ref. | - | - | Ref. | - | - |
|  |  | Pre-operative | 2.69 | 1.01–7.17 | 0.047* | 1.51 | 0.60–3.80 | 0.38 | 0.45 | 0.18–1.11 | 0.08 |
|  |  | Post-operative | 2.04 | 0.60–6.89 | 0.25 | 3.19 | 1.19–8.57 | 0.02* | 0.97 | 0.37–2.57 | 0.95 |
|  |  | Pre- and post-operative | 2.44 | 0.81–7.35 | 0.11 | 2.93 | 1.15–7.51 | 0.02* | 1.11 | 0.47–2.62 | 0.81 |
|  | Non-surgical treatment prior to surgery | None | Ref. | - | - | Ref. | - | - | Ref. | - | - |
|  |  | Laser | 1.40 | 0.39–5.01 | 0.60 | 0.63 | 0.24–1.68 | 0.36 | 0.82 | 0.30–2.29 | 0.71 |
|  |  | Air-flow | 1.69 | 0.39–7.33 | 0.48 | 0.56 | 0.18–1.76 | 0.32 | 0.67 | 0.20–2.22 | 0.51 |
|  |  | Chitosan brush | 0.63 | 0.05–7.66 | 0.72 | 0.32 | 0.01–6.85 | 0.46 | 0.38 | 0.02–8.41 | 0.54 |
|  |  | Mechanical debridement | 0.35 | 0.06–1.92 | 0.22 | 2.50 | 0.87–7.16 | 0.09 | 2.78 | 0.91–8.46 | 0.07 |
|  |  | Combined | 5.41 | 0.22–132.86 | 0.30 | 0 | 0 | 0 | 0 | 0 | 0 |
|  | Treatment approach | OFD | Ref. | - | - | Ref. | - | - | Ref. | - | - |
|  |  | Impl | 0.93 | 0.20–4.39 | 0.93 | 0.95 | 0.20–4.56 | 0.94 | 0.95 | 0.20–4.56 | 0.94 |
|  |  | Rec | 0.65 | 0.19–2.14 | 0.47 | 0.86 | 0.26–2.89 | 0.81 | 1.07 | 0.33–3.54 | 0.91 |
|  |  | Comb | 0.47 | 0.12–1.77 | 0.26 | 1.94 | 0.56–6.79 | 0.30 | 1.19 | 0.32–4.39 | 0.79 |
|  | Surgeon | Surgeon 0 | Ref. | - | - | Ref. | - | - | Ref. | - | - |
|  |  | Surgeon 1 | 1.27 | 0.43–3.72 | 0.67 | 0.46 | 0.16–1.31 | 0.15 | 0.40 | 0.13–1.24 | 0.11 |
|  |  | Surgeon 2 | 0.24 | 0.07–0.86 | 0.03* | 1.33 | 0.59–3.01 | 0.49 | 1.20 | 0.51–2.83 | 0.67 |
|  |  | Surgeon 3 | 1.35 | 0.47–3.88 | 0.58 | 0.65 | 0.26–1.62 | 0.35 | 0.98 | 0.41–2.34 | 0.96 |
| Reconstrucive therapy | Bone grafting material | Autogenous bone | Ref. | - | - | Ref. | - | - | Ref. | - | - |
|  |  | Deproteinized bovine bone mineral | 0.29 | 0.04–2.03 | 0.21 | 1.95 | 0.23–16.52 | 0.54 | 2.99 | 0.58–15.34 | 0.19 |
|  |  | Collagen-stabilized deproteinized bovine bone mineral | 0.61 | 0.12–3.18 | 0.56 | 0.56 | 0.08–4.04 | 0.56 | 1.24 | 0.29–5.28 | 0.77 |
|  |  | Deproteinized bovine bone mineral+ Autogenous bone | 1.12 | 0.22–5.74 | 0.89 | 3.67 | 0.65–20.80 | 0.14 | 1.24 | 0.29–5.21 | 0.77 |
|  |  | Collagen-stabilized deproteinized bovine bone mineral+ Autogenous bone | 1.31 | 0.16–10.56 | 0.80 | 5.07 | 0.76–33.62 | 0.09 | 0.19 | 0.01–4.45 | 0.30 |
|  | Membrane | No/Yes | 1.07 | 0.48–2.36 | 0.87 | 2.14 | 0.92–4.98 | 0.08 | 0.60 | 0.29–1.25 | 0.17 |
| Baseline |  | max PD | 0.78 | 0.66–0.91 | 0.002* | 1.13 | 0.97–1.30 | 0.11 | 1.27 | 1.09–1.47 | 0.002* |
|  |  | mean PD | 0.76 | 0.62–0.92 | 0.006* | 1.11 | 0.93–1.32 | 0.25 | 1.12 | 0.94–1.34 | 0.20 |
|  |  | KM | 0.90 | 0.75–1.08 | 0.25 | 0.87 | 0.73–1.03 | 0.11 | 0.99 | 0.84–1.17 | 0.92 |
|  |  | Sup | 0.60 | 0.21–1.67 | 0.33 | 3.57 | 1.47–8.72 | 0.005* | 1.55 | 0.60–4.03 | 0.37 |
|  |  | BOP | 0.64 | 0.21–1.96 | 0.43 | 1.17 | 0.40–3.45 | 0.77 | 1.00 | 0.34–2.93 | 0.99 |

Ref.: reference, -: not available, *: The difference is statistically significant. Variables with *p* < 0.05 in univariate screening were considered candidate predictors for multivariable modeling. For categorical predictors, all levels were modeled to preserve the full variable structure.
